# Detection of Vertical Root Fractures Using Cone-Beam Computed Tomography in the Presence and Absence of Gutta-Percha

**DOI:** 10.1155/2018/1920946

**Published:** 2018-07-09

**Authors:** Ehsan Hekmatian, Mitra Karbasi kheir, Hossein Fathollahzade, Mahnaz Sheikhi

**Affiliations:** ^1^Dental Implant Research Center, Department of Oral and Maxillofacial Radiology, School of Dentistry, Isfahan University of Medical Sciences, Isfahan, Iran; ^2^Department of Oral and Maxillofacial Radiology, School of Dentistry, Islamic Azad University, Isfahan (Khorasgan) Branch, Isfahan, Iran; ^3^Department of Oral and Maxillofacial Radiology, School of Dentistry, Isfahan University of Medical Sciences, Isfahan, Iran; ^4^Torabinejad Research Center, Department of Oral and Maxillofacial Radiology, School of Dentistry, Isfahan University of Medical Sciences, Isfahan, Iran

## Abstract

**Background:**

Vertical root fractures (VRFs) can significantly reduce dental prognosis. Cone-beam computed tomography (CBCT) offers better visualization of VRF than conventional radiography. However, gutta-percha creates artifacts in cone-beam CT (CBCT) images and reduces the diagnosis quality. The purpose of this study was to evaluate the accuracy of CBCT in detection of VRF in presence and absence of gutta-percha in canals.

**Materials and Methods:**

In this cross-sectional study, 50 extracted mandibular premolars were selected. After preparing the access cavity, canals were instrumented using step-back method, and gutta-percha #40 was placed afterwards. The fractures were created using electromechanical universal testing machine on 25 teeth. The teeth were randomly placed in dry mandibular dental sockets, and the first CBCTs were taken. Then the gutta-percha was removed, and the second CBCTs were taken.

**Results:**

The results of Kappa coefficient between two observers in roots with and without gutta-percha were 0.644 and 0.830, respectively (*p* value ≤ 0.001). The sensitivity and specificity of VRF diagnosis in assessing gutta-percha filled canals were 32% and 68% for the first observer, respectively (Kappa < 0.000,* p* value = 1.000), and 40% and 68% for the second observer, respectively (Kappa = 0.080,* p* value = 0.556). The sensitivity and specificity of VRF diagnosis in assessing the empty canals (without gutta-percha) were 72% and 96% for the first observer, respectively (Kappa = 0.680,* p* value ≤ 0/001), and 72% and 96% for the second observer, respectively (Kappa = 0.680,* p* value ≤ 0/001).

**Conclusion:**

The intracanal filling materials such as gutta-percha reduce the diagnostic ability of vertical root fractures. Hence, it is recommended to remove those materials from root canals before imaging to improve the diagnostic potential of CBCT.

## 1. Introduction

Vertical root fracture (VRF) occurs along the tooth axis and usually extends from the apex of root to the coronal part and from the internal wall of root canal to the root surface. VRFs may be complete (involving both sides of the root) or incomplete (involving one side of the root). The incidence of VRF in endodontically treated teeth is between 3.7 and 30.8%. VRF is more prevalent in premolars and mesial root of mandibular molars [1, 2]. VRF reduces tooth prognosis, can lead to inflammation, followed by bone resorption and granulation tissue formation, and can result in tooth extraction. Identification of VRF is challenging and requires the combination of clinical and radiographic signs and sometimes surgical findings. Overlap of adjacent structures in two-dimensional radiographs (conventional radiography) limits the visualization of fracture line. Computed tomography (CT) and cone-beam computed tomography (CBCT), on the other hand, can provide a better visualization of fracture line through multiplanar reconstructed images (axial, coronal, and sagittal planes). However, radiopaque materials like gutta-percha create artifacts in CBCT images due to beam hardening and reduce the diagnostic quality [3–11]. The purpose of this study was to evaluate the accuracy of CBCT in detection of longitudinal root fractures in presence and absence of gutta-percha in canals.

## 2. Materials and Methods

**Figure 1:**
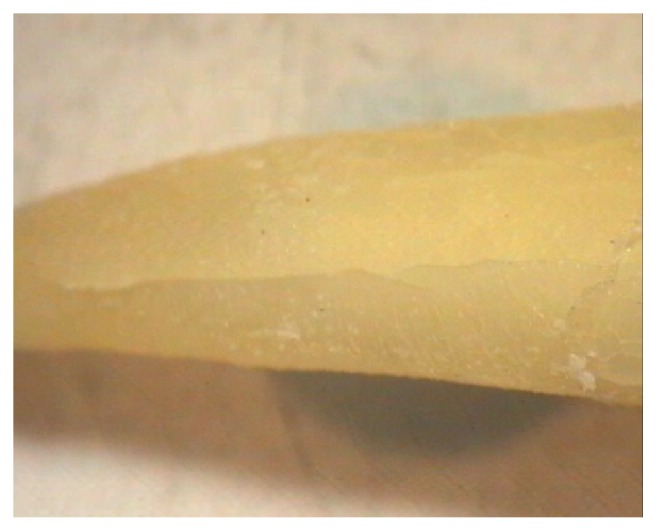
Vertical root fracture under stereomicroscope.

**Figure 2:**
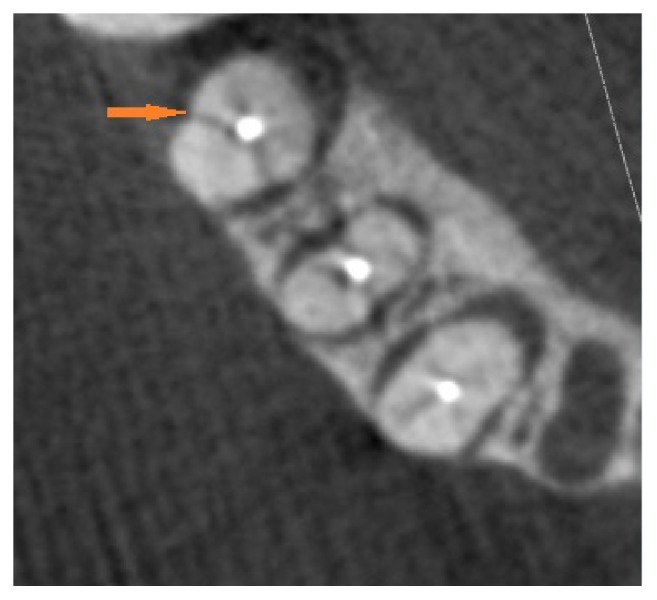
CBCT, axial view; red arrow: VRF in gutta-percha filled canals.

**Figure 3:**
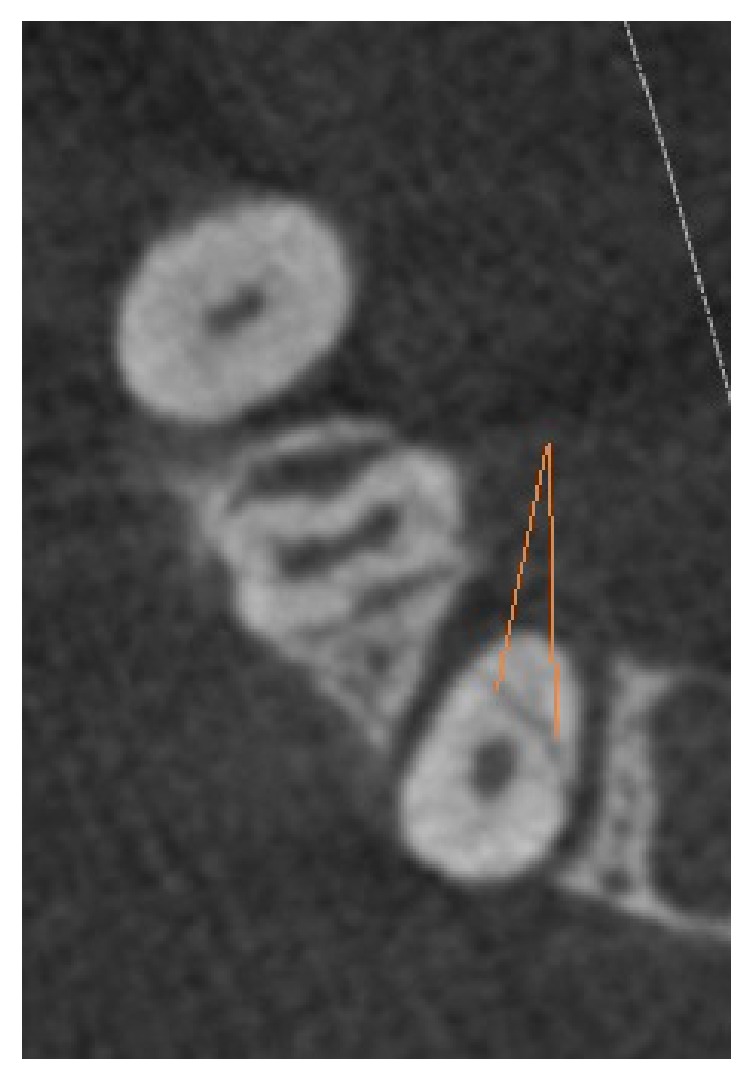
CBCT, axial view; red lines: VRF in empty canals (without gutta-percha).

In this cross-sectional study, 50 extracted mandibular premolars were selected and cleaned of calculus, soft tissue, and debris by hand instrumentation. After surface debridement, they were disinfected with 5.25% sodium hypochlorite (NaOCl). It took two months to collect the extracted teeth. All the teeth were prepared and restored by a single operator to prevent interoperator bias. Standard access cavities were made on them by a cylindrical diamond bur (Tiz Kavan Co., Iran), which was mounted on a high-speed headpiece, using air-water coolant spray (CH-4T5NSK B2/B3, Japan, A1101800). Every four cavities were prepared by a new bur to avoid crack formation. Then, all root canals were instrumented by a circumferential filing technique using hand K-files up to #40 file (Dentsply Maillefer, Ballaigues, Switzerland) using step-back canal preparation method. Then, a gutta-percha #40 was placed in each root canal. 25 teeth were included in the control group and 25 teeth were randomly selected for induction of root fracture. The fracture was artificially created using an electromechanical universal testing machine (k-21046, Walter+Bai, Switzerland) with cross-speed of 0.2 mm/min. The load increased to 500-700 N until fracture occurred and then the load was immediately stopped as shown by the diagram displayed on the system monitor. All fractured teeth were inspected by a stereomicroscope (Trinocular Zoom Stereo Microscope, SMP 200, HP, USA) at 10× magnification. Stereomicroscope was used as the gold standard to confirm the presence and direction of the fracture line (Figure 1). The other specimens in the control group were also assessed by stereomicroscope to assure that they were fracture-free. The teeth (with and without VRF) were randomly placed in dry mandibular dental sockets. For soft tissue reconstruction, the specimens were placed in a water container, and the first CBCT images were taken. Then the gutta-percha was carefully removed from all root canals to avoid changes in the fracture line/lines, and the second CBCT was taken. CBCT images were taken by Sirona Orthophos, GALILEOS version 1.7, XG 3D (Sirona, Germany). The exposure settings were 85 kVp, 13 mA, and 5.1 sec. The field of view (FOV) of the device was 5×5.5 cm. The images were analyzed by two maxillofacial radiologists on an LG LED computer viewer (E2042C, Korea) using Sidexis IX software. CBCT projections were analyzed in different sectional planes (tangential, cross-sectional, and axial) to detect the presence or absence of VRF, with the possibility to adjust the brightness and contrast and to use the zoom tool (Figures 2 and 3). The observers had been calibrated using pilot samples before evaluation of the main samples.

For statistical analysis, the data were analyzed by the Statistical Package for Social Sciences (SPSS) (version 22, SPSS Inc., Chicago, IL). Kappa coefficient was used to analyze the correlation between two observers and to assess the effect of gutta-percha presence on the diagnostic ability of observers.

## 3. Results

The results of Kappa coefficient between two observers in roots with and without gutta-percha were 0.644 and 0.830, respectively (*p* value ≤ 0.001).

The frequencies of VRF on CBCT images observed by the first and second observers in control and test groups in gutta-percha filled canals are shown in Table 1.

The sensitivity and specificity of VRF diagnosis for the first observer in assessing gutta-percha filled canals were 32% and 68%, respectively (Kappa < 0.000,* p* value = 1.000).

The sensitivity and specificity of VRF diagnosis for the second observer in assessing gutta-percha filled canals were 40% and 68%, respectively (Kappa = 0/080,* p* value = 0/556).

The frequencies of VRF on CBCT images observed by the first and second observers in control and test groups in empty canals (without gutta-percha) are shown in Table 2.

The sensitivity and specificity of VRF diagnosis for the first observer in assessing empty canals (without gutta-percha) were 72% and 96%, respectively (Kappa = 0/680,* p* value ≤ 0/001).

The sensitivity and specificity of VRF diagnosis for the second observer in assessing empty canals (without gutta-percha) were 72% and 96%, respectively (Kappa = 0/680,* p* value ≤ 0/001).

Assessment of VRF in empty canals (without gutta-percha) showed a significant correlation with control samples. However, there was a poor correlation between test and control groups in canals with gutta-percha.

## 4. Discussion

**Figure 4:**
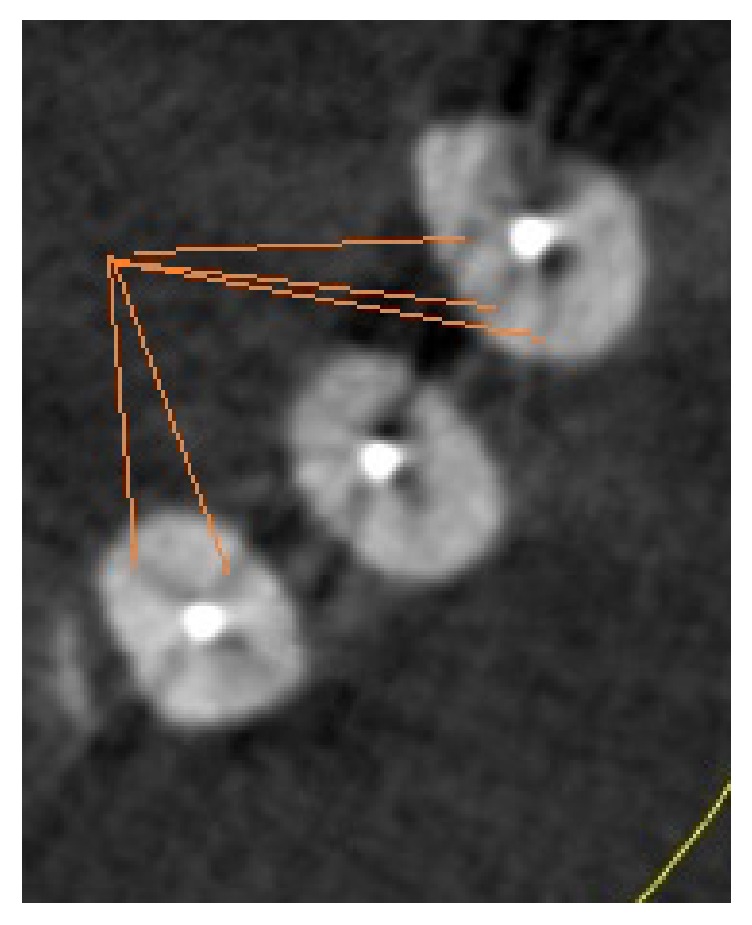
Effect of beam hardening on producing artifacts similar to VRF in gutta-percha filled canals.

Diagnosis of VRF is important for clinicians because clinic-radiographic signs are not completely diagnostic and are similar to other endodontic and periodontal diseases. Failure in VRF diagnosis may lead to surgical procedures or tooth extraction. In this in vitro study, the accuracy of CBCT in detection of VRF in gutta-percha root-filled canals and empty canals (without gutta-percha) was assessed. The presence of radiopaque materials in the root canals, due to beam hardening and creation of radiolucent and radiopaque lines around the radiopaque materials, reduces the diagnostic ability of CBCT [12]. The results of present study showed that the overall sensitivity and specificity of CBCT in detection of VRF in empty canals (without gutta-percha) were significantly higher than those of gutta-percha root-filled canals. In other words, when gutta-percha was placed within root canals, the ability of CBCT in diagnosing VRF was significantly reduced; therefore, gutta-percha can be considered an artifact creating factor (Figure 4).

Our findings were in accordance with those of Neves et al.'s study that evaluated the effects of different intracanal materials on the ability of CBCT in VRF detection and found that the presence of gutta-percha had a negative influence on the diagnostic ability of CBCT. In this study, the sensitivity of VRF diagnosis reduced when gutta-percha was present in the root canal [4]. Also, the findings confirmed the results of Likubo et al.'s study. Their results showed that gutta-percha caused artifacts similar to the fracture lines on CBCT. The difference between our study and that study was consideration of control group, which provided the possibility of measuring the negative effects of gutta-percha in the present study [13]. Further, the results of the present research were in agreement with those of Khedmat et al.'s study, in which the effect of gutta-percha on diagnosis of vertical root fracture was investigated. In the absence of gutta-percha, CBCT showed a higher diagnostic ability than conventional radiography or even CT scan. However, in the presence of gutta-percha, the diagnostic ability of CBCT was dramatically declined, which is similar to the results of the current study [14].

However, the findings of this study were not in agreement with those of Menezes et al.'s study that reported no significant differences in VRF detection between absence and presence of gutta-percha in the root canals [15]. The difference between the results of their study with those of the current research can be due to the fact that, in Menezes et al.'s study, the teeth with and without gutta-percha had no similar fractures because of the difference in degree and intensity of fracture. The degree and intensity of vertical root fracture, as confounding factors, can affect the diagnostic ability. In the present study, CBCT was performed on all the fractured and nonfractured teeth filled with gutta-percha. Then, gutta-percha was removed from all the root canals, and CBCT was carried out again to eliminate the effect of this confounding factor and ensure that the fracture lines remained constant in the teeth. In other words, in the present study, the degree and intensity of fractures were kept constant in fractured teeth in the presence and absence of gutta-percha. So, this study could be the first attempt to analyze the effect of intracanal filling materials on the diagnostic ability of VRF by eliminating the effect of degree and intensity of fracture as confounding factors.

## 5. Conclusion

The intracanal filling materials such as gutta-percha reduce the diagnostic ability of the vertical root fractures. Hence, it is recommended to remove those materials from root canals before imaging to improve the diagnostic potential of CBCT.

## Figures and Tables

**Table 1:** The frequencies of VRF on CBCT images observed by the first and second observers in control and test groups in gutta-percha filled canals.

Observer 1				Observer 2			
Groups	Test			Groups	Test		
Control	Variables	Fracture	No fracture	Control	Variables	Fracture	No fracture
	Fracture	8	17		fracture	10	15
	No fracture	8	17		No fracture	8	17

**Table 2:** The frequencies of VRF on CBCT images observed by the first and second observers in control and test groups in empty canals.

Observer 1				Observer 2			
Groups	Test			Groups	Test		
Control	Variables	Fracture	No fracture	Control	Variables	Fracture	No fracture
	Fracture	18	7		fracture	18	7
	No fracture	1	24		No fracture	1	24

## Data Availability

The data used to support the findings of this study are available from the corresponding author upon request.
